# Prognostic significance of early changes in serum biomarker levels in patients with newly diagnosed metastatic prostate cancer

**DOI:** 10.1038/s41598-019-48600-8

**Published:** 2019-08-19

**Authors:** Shintaro Narita, Kyoko Nomura, Shingo Hatakeyama, Masahiro Takahashi, Toshihiko Sakurai, Sadafumi Kawamura, Senji Hoshi, Masanori Ishida, Toshiaki Kawaguchi, Shigeto Ishidoya, Jiro Shimoda, Hiromi Sato, Koji Mitsuzuka, Tatsuo Tochigi, Norihiko Tsuchiya, Chikara Ohyama, Yoichi Arai, Kengo Nagashima, Tomonori Habuchi

**Affiliations:** 1 Department of Urology, Akita University School of Medicine, Akita, Japan; 2 Department of Environmental Health Science and Public Health, Akita University School of Medicine, Akita, Japan; 3 Department of Urology, Hirosaki University School of Medicine, Hirosaki, Japan; 4 Department of Urology, Tohoku University School of Medicine, Sendai, Japan; 5 Department of Urology, Yamagata University School of Medicine, Yamagata, Japan; 6 Department of Urology, Miyagi Cancer Center, Miyagi, Japan; 7 Department of Urology, Yamagata Prefectural Central Hospital, Yamagata, Japan; 8 Department of Urology, Iwate Prefectural Isawa Hospital, Isawa, Japan; 9 Department of Urology, Aomori Prefectural Central Hospital, Aomori, Japan; 10 Department of Urology, Sendai City Hospital, Sendai, Japan; 11 Research Center for Medical and Health Data Science, The Institute of Statistical Mathematics, Azabu, Japan; 12 Michinoku Japan Urological Cancer Study Group (MJUCSG), Sendai, Japan

**Keywords:** Prostate cancer, Tumour biomarkers

## Abstract

We evaluated the impact of early changes in serum biomarker levels on the survival of patients with metastatic hormone-sensitive prostate cancer (mHSPC) who were initially treated with androgen deprivation therapy (ADT). We retrospectively investigated 330 patients with mHSPC whose serum maker levels were at baseline and at 2–4 months. An optimal Cox regression model was established with the highest optimism-corrected concordance index based on 10-fold cross-validation. The median cancer-specific survival (CSS) and overall survival (OS) were 7.08 and 6.47 years (median follow-up, 2.53 years), respectively. In the final optimal Cox model with serum biomarker levels treated as time-varying covariates, prostate-specific antigen (PSA), hemoglobin (Hb), and alkaline phosphatase (ALP) significantly increased the risk of poor survival in the context of both CSS and OS. Kaplan–Meier curves stratified by the three risk factors of high PSA, low Hb and high ALP desmondtated that median OS were not reached with none of these factors, 6.47 years with one or two factors, and 1.76 years with all three factors.Early changes in serum biomarker levels after ADT may be good prognostic markers for the survival of patients with mHSPC.

## Introduction

Androgen deprivation therapy (ADT) is considered to be a mainstay of initial treatment for newly diagnosed metastatic hormone-sensitive prostate cancer (mHSPC). However, castration resistance is inevitable, and most patients with metastatic prostate cancer die of cancer progression^1^. Recently, large randomized trials demonstrated a significant overall survival (OS) benefit with additional upfront docetaxel and abiraterone acetate treatment in these patients^2,3^. Previous reports have shown that a percentage of patients with newly diagnosed mHSPC require early intervention with other medications along with ADT, suggesting the importance of both recharacterization and subcategorization in patients with mHSPC who are initially treated with ADT monotherapy.

Previous studies have proposed several candidate biomarkers and risk stratification models for patients with mHSPC treated with ADT^4,5^. Moreover, recent studies have shown that dynamic changes in serum biomarker levels at the early “on therapy” period, including alterations in the levels of prostate-specific antigen (PSA) and alkaline phosphatase (ALP), are strong prognostic factors for clinical outcomes, including survival, in patients with mHSPC and/or castration-resistant prostate cancer (CRPC)^6–8^. However, there is still less evidence regarding the impact of early changes in serum biomarker levels on clinical outcomes after ADT in recent diagnosed patients with mHSPC.

Therefore, this study aimed to investigate the prognostic impact of serum biomarker levels during early stages of ADT on the survival and develop a prognostic model for deployment among patients with newly diagnosed mHSPC.

## Results

Table 1 shows the patients’ characteristics. For the 330 patients analyzed, the median age was 72 years (interquartile range, 65–78 years). Approximately 90.9%, 58.5%, and 9.7% of patients had bone, lymph node, and visceral metastases, respectively. A combined androgen blockade was employed in 78.5% of the patients, whereas 21.5% were treated using LHRH antagonist.

**Table 1:** Patient characteristics.

Variables		n = 330
Patient characteristics at diagnosis		
Age, y, median (IQR)		72 (65–78)
BMI, kg/M^2^, median (IQR)		22.7 (20.5–24.6)
ECOG-PS, No. (%)	0	177 (53.6)
	≥1	153 (46.4)
Baseline PSA level, ng/ml, median (IQR)		345.5 (96.7–930.0)
Baseline Hb level, g/dl, median (IQR)		13.1 (11.9–14.3)
Baseline ALP level, IU, median (IQR)		405.0 (267.0–911.7)
Baseline LDH level, IU, median (IQR)		215.5 (184.0–265.0)
Baseline Alb level, md/dl, median (IQR)		4.0 (3.7–4.3)
Biopsy Gleason Score, No. (%)	≤8	167 (50.6)
	≥9	163 (49.4)
Site of metstasis, No. (%)	Bone	300 (90.9)
	Lymph node	193 (58.5)
	Visceral	32 (9.7)
	Presence of bone pain, No. (%)	160 (48.5)
EOD score, No. (%)		
	0	30 (9.1)
	1	99 (30.0)
	2	89 (27.0)
	3	80 (24.2)
	4	32 (9.7)
Initial hormonal therapy		
LHRH agonist + anti-androgen		204 (61.8)
LHRH antagonist + anti-androgen		37 (11.2)
LHRH antagonist monotherapy		34 (10.3)
LHRH agonist monotherapy		9 (2.7)
Orchidectomy + anti-androgen		18 (5.5)
Orchidectomy		10 (3.0)
Others		18 (5.5)
Serum markers measured at 2–4 months after initial hormonal therapy		
PSA level at 2–4 months, ng/ml, median (IQR)		3.1 (0.5–18.3)
Hb level at 2–4 months, g/dl, median (IQR)		12.4 (11.8–13.4)
ALP level at 2–4 months, IU, median (IQR)		392.5 (273.0–680.0)
LDH level at 2–4 months, IU, median (IQR)		213.0 (186.0–242.0)
Albumin level at 2–4 months, mg/dl, median (IQR)		4.2 (3.9–4.5)

IQR: Interquartile range; BMI: body mass index; ECOG-PS: Eastern Cooperative Oncology Group- Peformance Status; PSA: prostate specific antigen; Hb: hemoglobin; ALP: alkaline phosphatase; LDH: lactate dehydrogenase; EOD: extent of bone disease; LHRH: Luteinizing Hormone-Releasing Hormone.

During the median 2.53-year follow-up, 111 (33.6%) patients died, of which 87 (26.4%) died due to progressive prostate cancer. The median cancer-specific survival (CSS) and OS were 7.08 and 6.47 years, respectively.

Table 2 shows the univariable analysis for CSS and OS in patients with mHSPC treated with ADT. In both Cox models for CSS and OS, decreased body mass index (BMI) (*p* = 0.026 and *p* = 0.002), Gleason score of ≥9 (*p* = 0.035 and *p* = 0.043), extent of bone metastasis (EOD) score of ≥2 (both *p*s < 0.001), low Hb levels (*p* = 0.008 and *p* = 0.004 at baseline and *p* = 0.002 and *p* = 0.001 at 2–4 months) and high ALP levels (*p* = 0.023 and *p* = 0.046 at baseline and both *p*s < 0.001 at 2–4 months), high lactate dehydrogenase (LDH) level at baseline (both *p*s < 0.001), and high PSA level at 2–4 months (both *p*s < 0.001) were shown to be significant risk factors for worse survival. In addition, lymph node metastasis was an independent risk factor for poor CSS (*p* = 0.013).

**Table 2:** Univariable analysis for cancer specific survival and overall survival in patients with mHSPC treated with ADT.

		Cancer-specific survival			Overall survival		
		HR	95%CI	p value	HR	95%CI	p value
Medical institute	Continuous	0.95	0.87–1.04	0.241	0.95	0.88–1.03	0.191
Year of initial hormonal therapy	Continuous	1.01	0.91–1.11	0.893	0.99	0.90–1.08	0.749
Patient characteristics at diagnosis							
Age	Continuous	0.99	0.97–1.02	0.527	1.01	0.99–1.03	0.426
BMI, kg/m^2^	Continuous	0.92	0.86–0.99	0.026	0.91	0.85–0.96	0.002
ECOG-PS	≥1 vs. 0	1.24	0.81–1.90	0.318	1.33	0.91–1.93	0.141
Gleason Score	≥9 vs. ≤8	1.58	1.03–2.42	0.035	1.47	1.01–2.14	0.043
Site of metstasis							
Bone	(+) vs. (−)	1.67	0.68–4.12	0.266	1.51	0.70–3.24	0.295
Lymph node	(+) vs. (−)	1.79	1.13–2.83	0.013	1.47	0.99–2.18	0.054
Visceral	(+) vs. (−)	0.72	0.31–1.64	0.428	0.95	0.50–1.83	0.884
Presence of bone pain	(+) vs. (−)	1.29	0.84–1.97	0.240	1.21	0.83–1.75	0.326
EOD score	≥2 vs. ≤1	2.62	1.59–4.32	<0.0001	2.23	1.46–3.41	<0.0001
Initial hormonal therapy							
LHRH antagonist	(+) vs. (−)	1.75	0.99–3.09	0.055	1.61	0.96–2.70	0.070
Serum markers							
Baseline							
PSA, ng/mL (345< vs. ≤345)	345< vs. ≤345	1.00	0.66–1.53	0.988	0.98	0.67–1.42	0.897
Hb, g/dL (≤12 vs. 12<)	≤12 vs. 12<	1.82	1.17–2.82	0.008	1.77	1.20–2.62	0.004
ALP, IU (350< vs. ≤350)	350< vs. ≤350	1.68	1.07–2.63	0.023	1.49	1.01–2.19	0.046
LDH, IU (220< vs. ≤220)	220< vs. ≤220	2.28	1.48–3.51	<0.0001	2.19	1.50–3.21	<0.0001
Albumin, mg/dL (≤3.5 vs. 3.5<)	≤3.5 vs. 3.5<	1.52	0.87–2.65	0.144	1.60	0.99–2.61	0.057
2–4 months after initial therapy							
PSA, ng/mL (3.1< vs. ≤3.1)	3.1< vs. ≤3.1	3.05	1.93–4.81	<0.0001	2.32	1.57–3.42	<0.0001
Hb, g/dL (≤12 vs. 12<)	≤12 vs. 12<	1.97	1.28–3.04	0.002	1.93	1.31–2.83	0.001
ALP, IU (350< vs. ≤350)	350< vs. ≤350	2.62	1.62–4.22	<0.0001	2.59	1.70–3.94	<0.0001
LDH, IU (220< vs. ≤220)	220< vs. ≤220	1.15	0.74–1.79	0.526	1.30	0.89–1.90	0.180
Albumin, mg/dL (≤3.5 vs. 3.5<)	≤3.5 vs. 3.5<	1.94	0.84–4.47	0.121	2.03	0.98–4.19	0.056

ALP: alkaline phosphatase; BMI: body mass index; ECOG-PS: Eastern Cooperative Oncology Group- Peformance Status; EOD: extent of bone disease; PSA: prostate specific antigen; Hb: hemoglobin; LDH: lactate dehydrogenase; LHRH: Luteinizing Hormone-Releasing Hormone; HR: hazard ratio; 95% CI: 95% confidence interval.

**Figure 1:**
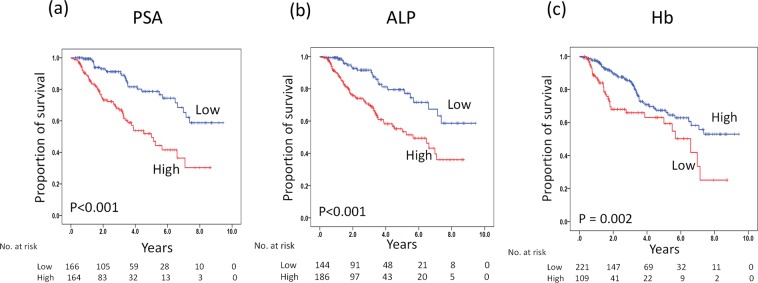
Kaplan–Meier curves for CSS in patients with mHSPC who were initially treated with ADT. CSS stratified by PSA (**a**), ALP (**b**), and Hb (**c**) at 2–4 months, respectively. The *p* values were computed using a log-rank test.

**Figure 2:**
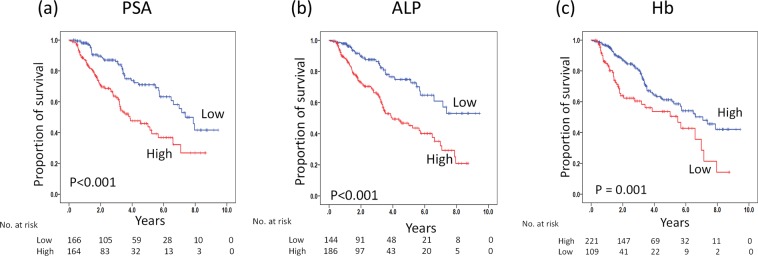
Kaplan–Meier curves for OS in patients with mHSPC who were initially treated with ADT. OS stratified by PSA (**a**), ALP (**b**), and Hb (**c**) at 2–4 months, respectively. The *p* values were computed using a log-rank test.

The Kaplan–Meier curves of each binary serum biomarker PSA, ALP, and Hb at 2–4 months post-ADT are shown in Fig. 1 for CSS and in Fig. 2 for OS. The median CSS in patients with high PSA, high ALP, or low Hb levels at 2–4 months was shorter than that in patients with low PSA, low ALP, or high Hb levels (log-rank, *p* < 0.001, <0.001, and = 0.002, respectively). Additionally, the median OS in patients with high PSA, high ALP, or low Hb levels at 2–4 months was significantly shorter than that in patients with low PSA, low ALP, or high Hb levels at 2–4 months (log-rank, *p* < 0.001, <0.001, and = 0.001, respectively).

Table 3 shows the model selection process with time-varying covariates of PSA, Hb, ALP, LDH, and albumin based on 10-fold cross-validation. Upon entering all significant variables selected at *p* < 0.2 in a univariate model into multivariable models, we identified the top five models for CCS and OS based on the observation of higher numbers of concordance indices (*C*-indices) (Table 3). Among these, the final Cox model for CSS and OS was 0.771 with lymph node metastasis, EOD score, PSA, Hb, and ALP and 0.732 with BMI, Gleason score, lymph node metastasis, PSA, Hb, and ALP, respectively. These final Cox models demonstrated that *p* values for the proportional hazards score test^9^ of selected variables calculated were all >0.05, indicating no statistical significant violation of the proportional hazard assumption. We also ran 10-fold cross-validation by using baseline serum biomarker levels instead of time-varying covariates of these biomarker levels. The highest *C*-indices shown in Supplementary Table 1 were all lower than those in the model with time-varying covariates (Table 3). Therefore, the Cox model with serum marker levels measured at 2–4 months as time-varying covariates had a better predictive ability than the model with serum marker levels measured at baseline.

**Table 3:** Model selection with time-varying covariates of PSA, Hb, ALP, LDH, albumin based on 10-fold cross validation.

Outcome	Rank	Corrected *C*-index (10-fold CV)	Covariate	P-value for proportional hazards assumption*
Cancer-specific survival	1	0.771	Lymph node metastasis, EOD score, PSA, Hb, ALP	0.321
	2	0.770	BMI, Gleason score, Lymph node metastasis, EOD score, PSA, Hb	0.256
	3	0.770	BMI, Gleason score, Lymph node metastasis, EOD score, PSA, ALP	0.061
	4	0.770	Lymph node metastasis, EOD score, LHRH antagonist, PSA, Hb, ALP	0.230
	5	0.769	Lymph node metastasis, EOD score, PSA, Hb, ALP, Alb	0.229
Overall survival	1	0.732	BMI, Gleason score, Lymph node metastasis, PSA, Hb, ALP	0.193
	2	0.729	BMI, Gleason score, Lymph node metastasis, PSA, Hb, ALP, Alb	0.220
	3	0.726	BMI, Gleason score, Lymph node metastasis, LHRH antagonist, PSA, Hb, ALP	0.155
	4	0.726	BMI, Gleason score, Lymph node metastasis, LHRH antagonist, PSA, Hb, ALP, LDH	0.125
	5	0.725	BMI, Gleason score, Lymph node metastasis, PSA, Hb, ALP, LDH	0.150

*P-values for global null hypothesis based on weighted residual proposed by Grambsch & Therneau.

ALP: alkaline phosphatase; BMI: body mass index; EOD: extent of bone disease; Hb: hemoglobin; LDH: lactate dehydrogenase; LHRH: Luteinizing Hormone-Releasing Hormone; PSA: prostate specific antigen.

The final Cox regression models for CSS and OS are shown in Table 4. The optimal models demonstrated that the time-varying covariates of PSA, Hb, and ALP significantly increased the risk of poor survival for both CSS and OS—i.e., >3.1 ng/mL of PSA [hazard ratio (HR): 2.47; 95% confidence interval (CI): 1.55–3.94 and HR: 1.88; 95% CI: 1.25–2.82, respectively], >350 IU/l of ALP (HR: 1.87; 95% CI: 1.13–3.08 and HR: 2.03; 95% CI: 1.30–3.17, respectively), and ≤12 g/dL of Hb (HR: 1.86; 95% CI: 1.20–2.90 and HR: 1.86; 95% CI: 1.25–2.76, respectively) measured at 2–4 months after ADT significantly increased the risk of poor survival. In the final model for CSS, in addition to PSA, Hb, and ALP, the presence of lymph node metastasis (HR: 1.61; 95% CI: 1.01–2.58) and the EOD score with ≥2 (HR: 2.02; 95% CI: 1.20–3.39) were also significantly associated with poor survival. When the measurement time for time-varying covariates was limited to between 70 and 110 days, the final Cox models with the highest *C*-index, which satisfied the proportional hazard assumption, suggested an influence of lymph node metastasis, PSA, Hb, and ALP for CSS (*C*-index: 0.774) and that of PSA, Hb, ALP, and albumin for OS (*C*-index: 0.753). Both the models demonstrated that PSA, Hb, and ALP were all significantly associated with poor CSS and OS. These sensitivity analysis results are further presented in Supplementary Tables 2 and 3.

**Table 4:** Final Cox regression models for cancer-specific survival and overall survival in patients with mHSPC treated with ADT.

		Cancer-specific survival			Overall survival		
		HR	95% CI	P-value	HR	95% CI	P-value
Patient characteristics at diagnosis							
BMI, kg/m^2^	Continuous	—	—	—	0.97	0.91–1.03	0.349
Gleason score	≥9 vs. ≤8	—	—	—	1.33	0.91–1.95	0.140
Lymph node metastasis	(+) vs. (−)	1.61	1.01–2.58	0.046	1.37	0.91–2.05	0.129
EOD score	≥2 vs. ≤1	2.02	1.20–3.39	0.008	—	—	—
Serum makers (time-varying covariate)							
PSA, ng/mL	>3.1 vs. ≤3.1	2.47	1.55–3.94	<0.001	1.88	1.25–2.82	0.002
Hb, g/dL	≤12 vs. 12<	1.86	1.20–2.90	0.006	1.86	1.25–2.76	0.002
ALP, IU	>350 < vs. ≤350	1.87	1.13–3.08	0.014	2.03	1.30–3.17	0.002

ALP: alkaline phosphatase; BMI: body mass index; EOD: extent of bone disease; Hb: hemoglobin; PSA: prostate specific antigen.

**Figure 3:**
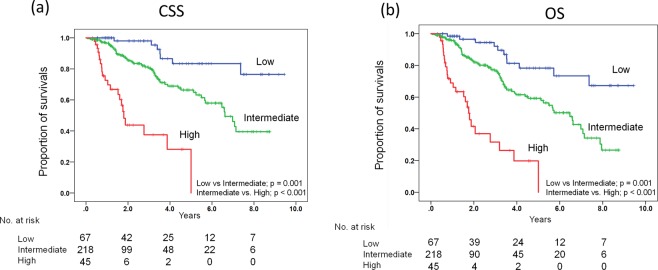
Kaplan–Meier curves for CSS (**a**) and OS (**b**) in patients with mHSPC who were initially treated with ADT according to the risk classification based on the presence of high serum levels of PSA and ALP at 2–4 months and a low serum level of Hb at 2–4 months. The risk groups were formed based on the combination of these three factors, as follows: zero risk factors, one or two risk factors, and three risk factors, respectively. The *p* values were computed using a log-rank test.

According to the final Cox models for CSS and OS, the derived prognostic risk factors in common were high PSA and ALP levels and low Hb level at 2–4 months. Therefore, the risk groups were formed based on combinations of these three factors, with patients having zero, one or two, and three risk factors defined as the low-, intermediate-, and high-risk groups, respectively. The Kaplan–Meier curves and median CSS and OS were subsequently calculated for these groups, and the results are shown in Fig. 3. The patients in the intermediate-risk group demonstrated significantly lower median CSS and OS than those in the low-risk group (for CSS, 6.62 years for intermediate-risk vs. not reached years for low-risk, *p* = 0.001; for OS, 6.47 years for intermediate-risk vs. not reached years for low-risk, *p* = 0.001). Additionally, patients in the high-risk group had significantly shorter median CSS and OS than those in the intermediate-risk group (for CSS, 1.80 years for high-risk vs. 6.62 years for intermediate-risk, *p* < 0.001; for OS, 1.76 years for high-risk vs. 6.47 years for intermediate-risk, *p* < 0.001). These results indicate that the risk stratification depending on the three risk factors with serum levels of PSA, Hb, and ALP at 2–4 months contributes to differences in the survival of patients with mHSPC.

## Discussion

Various PSA-related “on therapy” biomarkers have been reported in mHSPC^10^. In PSA kinetic variables, PSA nadir and time to PSA nadir are well-known risk factors for outcomes in mHSPC^11,12^. In recent years, several studies demonstrated that PSA level ≤0.2 ng/mL at 7 months was a strong prognostic factor for longer OS in patients with ADT^13,14^. These results suggested that “on therapy” PSA-related biomarker levels were promising candidates for predicting treatment outcomes in patients with mHSPC. However, it was implied that the prognosis can be predicted more than half a year after ADT initiation. In recent phase III trials showing the superiority of upfront docetaxel and abiraterone acetate administration for mHSPC, ADT was commenced within 120 days before randomization^2,3^. Therefore, it may be reasonable to predict patients with poor prognosis at initial stages (up to 3 or 4 months) and detect very early changes in serum biomarker levels after ADT initiation. Park *et al*. reported that shorter PSA half-time calculated as log 2 divided by the slope of the linear regression of log PSA versus the time using pre- and post-treatment PSA levels assayed were independent risk factors for poor CSS in patients with mHSPC^7^. In our study cohort, PSA levels at 2–4 months increased from baseline in several patients (data not shown), indicating that it is not feasible to calculate values from chronological values, including PSA half-time, for such patients. Altogether, evaluation of absolute PSA levels after ADT may be a useful option for predicting poor prognosis as it is simple and can be applied to all patients.

Regarding the “on therapy” non-PSA serum biomarker levels, a decline in Hb levels after 3 months of ADT was independently associated with shorter survival (HR, 1.10 per 1 g/dL decline; p = 0.0035) and shorter progression-free survival (HR, 1.08 per 1 g/dL decline; p = 0.013) after adjusting for potential confounders, including baseline Hb levels in 827 of 1,286 patients enrolled in the SWOG Study S8894 (Intergroup Study 0105)^15^. Consistent with these findings, the current study showed that Hb levels at 2–4 months represented a strong risk factor for CSS and OS. Moreover, serum ALP at 2–4 months was also an independent prognostic factor for CSS and OS in the present study. A previous study suggested that increased ALP levels at 12 weeks is a promising biomarker in bone metastatic CRPC patients treated with abiraterone acetate^8^. Although further validation study should be performed in the future, our study represents the first proposal of the impact of early changes in serum Hb and ALP levels in patients with mHSPC.

Several limitations of the current study should be noted. First, we focused on patients whose serum data at an early period after ADT were available, thus indicating that we excluded patients who died at a very early period. In the entire study cohort, although only 21 (3.5%) of 605 patients died within 3 months, a selection bias based on this fact cannot be avoided. Second, our study did not consider the impact of sequential treatments after initial ADT. Although the treatment duration was not statistically associated with CSS and OS in the current study, sequential treatments after ADT failure may play a role in the patient’s outcome. Furthermore, the cohort did not include patients who were treated with upfront docetaxel and abiraterone acetate, which are now considered to be the standard treatment for high-volume mHSPC. Novel risk factors for patients receiving upfront treatments should be elucidated in future studies. Finally, a retrospective study design and short follow-up duration were other limitations. Therefore, future studies with a longer follow-up period using a validation dataset are warranted.

In conclusion, high levels of PSA and ALP and low levels of Hb at 2–4 months are promising early “on therapy” prognostic biomarkers for survival in patients with newly diagnosed mHSPC who are treated with only ADT. Patients can be divided into different risk groups depending on the early changes in PSA, Hb, and ALP levels. Not only pretreatment risk factors but also early changes in serum biomarker levels may be useful for predicting poor survival in patients who require more aggressive treatment, including upfront chemotherapy and novel anti-androgens.

## Methods

### Patients

This retrospective multicenter study was conducted at nine medical institutes, in the Tohoku region, Japan. A consecutive group of 629 adult patients diagnosed with mHSPC between March 2008 and May 2016 was retrospectively identified at each institute. All patients initially received ADT, which consisted of orchiectomy, luteinizing hormone-releasing (LHRH) agonists/antagonists, alone or combined with bicalutamide. No patient received upfront docetaxel and/or abiraterone acetate as an initial therapy. Sequential treatments were administered after first-line ADT at the physician’s discretion.

The study was conducted in accordance with the Helsinki Declaration. The study was also approved by each institute’s ethical committee (Ethical committees of Akita University School of Medicine, Hirosaki University School of Medicine, Tohoku University School of Medicine, Yamagata University School of Medicine, Miyagi Cancer Center, Yamagata Prefectural Central Hospital, Iwate Prefectural Isawa Hospital, Aomori Prefectural Central Hospital, Sendai City Hospital). We applied an opt-out methodology is to provide accessible information to all patients to facilitate informed consent without interfering with the medical consultation^16^ and patients were informed of their inclusion in the study and were provided information on the institution’s website.

### Variables

The variables in the data set contained patients’ characteristics at the time of their diagnosis, including age; BMI (kg/m^2^); medical institute; years of diagnosis; years of initial ADT; Eastern Cooperative Oncology Group performance status score (ECOG-PS); biopsy Gleason score; site of metastasis (visceral, lymph node, or bone); presence of bone pain; EOD score; types of initial hormonal therapy; levels of serum biomarker PSA, Hb, ALP, LDH, and albumin; and date of cause-specific death and all-cause death. ECOG-PS and the presence of bone pain were evaluated by inquiry and physical examination. EOD scores for each patient were classified using bone scintigraphy at the time of the initial diagnosis according to the definition of Soloway *et al*.^17^. To investigate the changes in serum biomarker levels in the early phase after ADT, we defined 2*–*4 months (70*–*144 days) as the early phase.

### Database

**Figure 4:**
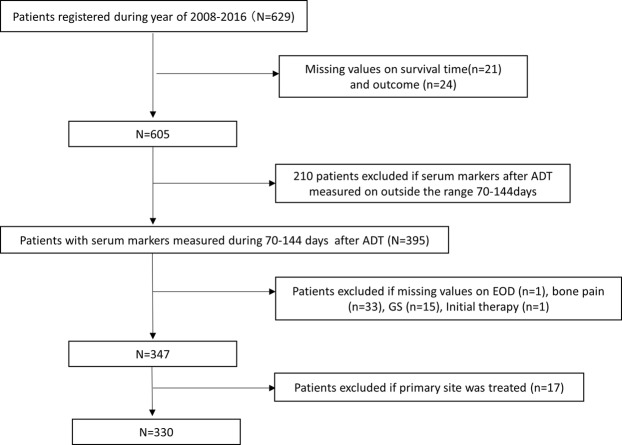
Scheme of patient selection. A consecutive group of 629 adult patients diagnosed with mHSPC between March 2008 and May 2016 was retrospectively identified at each institute. We first excluded 24 patients due to missing data regarding survival outcome and then excluded 275 patients due to missing data for variables required for the analyses. The remaining 330 patients were used as the study subjects for our analyses.

The study enrollment is shown in Fig. 4. We first excluded 24 patients due to missing values on survival outcome. We also excluded 210 patients because they had serum biomarker levels evaluated beyond the specified period of 2*–*4 months. For the remaining 395 patients, we interpolated the missing serum marker levels using an average value of each marker according to each cohort (medical institute). We interpolated BMI from the regression slope on age after stratification of cohorts. Finally, we excluded the missing values of EOD score (*n* = 1), bone pain (*n* = 33), Gleason score (*n* = 15), initial therapy (*n* = 1), and 17 patients whose primary site was treated, because we assumed that these variables were not interpolated for ethical considerations. The remaining 330 patients comprised the subjects in our analyses.

### Statistical analyses

The outcome of the present study included CSS and OS, which were calculated as the time from the diagnosis of mHSPC to death from prostate cancer or from any other cause. Patients known to be alive or lost to follow-up on the date of last contact were censored. Baseline characteristics were summarized using descriptive statistics (median and range for continuous variables, number and percentage for categorical variables). PSA level was divided at the median of its distribution at baseline (>345 ng/mL vs. ≤345 ng/mL) and at 2*–*4 months (>3.1 ng/mL vs. ≤3.1 ng/mL), otherwise the serum biomarkers were all divided into binary groups according to the normal ranges for Japanese males^18–21^: Hb (≤12 g/dL vs. >12 g/dL<), ALP (>350 IU vs. ≤350 IU), LDH (>220 IU vs. ≤220 IU), and albumin (≤3.5 mg/dL vs. >3.5 mg/dL).

The Kaplan–Meier method was applied to depict the CSS and OS curves between the binary groups of serum biomarkers at 2*–*4 months, and the comparison was statistically analyzed using a log-rank test. To investigate the impact of serum biomarker levels in the early phase after ADT initiation and to identify independent prognostic factors for CSS and OS, we applied a Cox proportional hazard model to calculate the HRs and 95% CIs. While building a Cox regression model, we considered PSA, Hb, ALP, LDH, and albumin levels measured at 2–4 months as time-varying variables because the levels of these markers were not constant over the follow-up duration. In order to select an optimal Cox model, while adjusting for all variables selected at *p* < 0.2 in the univariate models, we ran the 10-fold cross-validation. The highest optimism-corrected *C*-index value, which ranges from 0 to 1, can be used to measure and compare the discriminative power of prediction models^22^. Finally, we checked whether the best model chosen met the assumptions of the proportional hazard model based on the score test proposed by Grambsch and Therneau^9^. We ran these analyses while including serum biomarker levels measured at baseline and at 2–4 months, respectively, to compare which model has higher *C*-index values. Finally, we also conducted sensitivity analyses if the results were found to be robust when the period of time for time-varying covariates to be measured was redefined as 70 to 110 days.

Statistical analyses were performed using SPSS ver. 19.0 (IBM Corp., Armonk, NY, USA), SAS ver. 9.4 (SAS Institute Inc., Cary, NC, USA), and R ver. 3.4.1 (R Foundation for Statistical Computing, Vienna, Austria), with *p* < 0.05 considered to be statistically significant.

## Supplementary information

supplementary
