# Supplementary material for: Prognostic significance of early changes in serum biomarker levels in patients with newly diagnosed metastatic prostate cancer
Source: Sci Rep. 2019 Aug 19;9:12071. doi: 10.1038/s41598-019-48600-8 (PMC6700107; doi:10.1038/s41598-019-48600-8)
Supplement: Supplementary file 1 — supplementary [file 41598_2019_48600_MOESM1_ESM.docx]

Supplementary information

Title: Prognostic significance of early changes in serum biomarker levels in patients with newly diagnosed metastatic prostate cancer

Authors: Shintaro Narita, Kyoko Nomura, Shingo Hatakeyama, Masahiro Takahashi, Toshihiko Sakurai, Sadafumi Kawamura, Senji Hoshi, Masanori Ishida, Toshiaki Kawaguchi, Shigeto Ishidoya, Jiro Shimoda, Hiromi Sato, Koji Mitsuzuka, Tatsuo Tochigi, Norihiko Tsuchiya, Chikara Ohyama, Yoichi Arai, Kengo Nagashima, Tomonori Habuchi
